# Exercise-Induced Fatigue and Caffeine Supplementation Affect Psychomotor Performance but Not Covert Visuo-Spatial Attention

**DOI:** 10.1371/journal.pone.0165318

**Published:** 2016-10-21

**Authors:** Charlotte J. W. Connell, Benjamin Thompson, Gustav Kuhn, Nicholas Gant

**Affiliations:** 1 Exercise Neurometabolism Laboratory, Centre for Brain Research, University of Auckland, Auckland, New Zealand; 2 School of Optometry and Vision Science, University of Waterloo, Waterloo, Canada; 3 School of Optometry and Vision Science, University of Auckland, Auckland, New Zealand; 4 Department of Psychology, Goldsmiths, University of London, London, United Kingdom; Tokai University, JAPAN

## Abstract

Fatigue resulting from strenuous exercise can impair cognition and oculomotor control. These impairments can be prevented by administering psychostimulants such as caffeine. This study used two experiments to explore the influence of caffeine administered at rest and during fatiguing physical exercise on spatial attention—a cognitive function that is crucial for task-based visually guided behavior. In independent placebo-controlled studies, cohorts of 12 healthy participants consumed caffeine and rested or completed 180 min of stationary cycling. Covert attentional orienting was measured in both experiments using a spatial cueing paradigm. We observed no alterations in attentional facilitation toward spatial cues suggesting that covert attentional orienting is not influenced by exercise fatigue or caffeine supplementation. Response times were increased (impaired) after exercise and this deterioration was prevented by caffeine supplementation. In the resting experiment, response times across all conditions and cues were decreased (improved) with caffeine. Covert spatial attention was not influenced by caffeine. Together, the results of these experiments suggest that covert attentional orienting is robust to the effects of fatiguing exercise and not influenced by caffeine. However, exercise fatigue impairs response times, which can be prevented by caffeine, suggesting that pre-motor planning and execution of the motor responses required for performance of the cueing task are sensitive to central nervous system fatigue. Caffeine improves response time in both fatigued and fresh conditions, most likely through action on networks controlling motor function.

## Introduction

Prolonged, fatiguing exercise challenges cerebral homeostasis [1, 2] resulting in brain-based functional impairments, such as reduced output from the primary motor cortex [3]. This neural component of exercise fatigue, otherwise referred to as central fatigue, is poorly understood, but may develop because of impaired synthesis and metabolism of several neurotransmitters, including dopamine and noradrenaline [4–6]. In a recent study, we discovered that exercise fatigue reduces the velocity of saccadic eye movements [7] and is therefore capable of influencing the neural processes that support oculomotor control. Given that the oculomotor control of saccadic eye movements is closely related to the spatial orienting of visual attention, it is plausible that exercise fatigue may also challenge the attentional processes supporting saccade control, such visuospatial attention. Interestingly, when caffeine was supplemented during exercise, impairments in saccadic eye movement velocity were absent [7]. We propose that this effect is most likely due to the stimulant action of caffeine on central neurotransmission via adenosine antagonism, which results in up regulation of dopamine and heightened synthesis and turnover rates of noradrenaline [8].

The oculomotor control of saccadic eye movements is related to the spatial orienting of attention [9], a process that involves multiple stages of visual processing [10]. At its most basic level the oculomotor system orients the eyes to foveate information of interest and therefore contributes to overt attentional orienting. Attentional orienting can also occur without an eye movement towards the attended location; a process known as covert attentional orienting. Although the extent to which overt and covert attentional orienting are linked is uncertain [11, 12], the brain areas involved in shifting covert attention overlap considerably with those responsible for overt shifts of attention [9]. It is therefore possible that both forms of attentional orienting are vulnerable to the central effects of fatigue. This is important because efficient shifting of attention is crucial for effective processing of visual information [13–15] and therefore fatigue-induced deficits in attention may affect sports performance and compromise safe levels of visual surveillance. In this context, investigating the effect of exercise fatigue on covert attentional orienting is particularly important because any effect of fatigue would be independent from exercise-induced deficits in oculomotor control [7].

**Fig 1:**
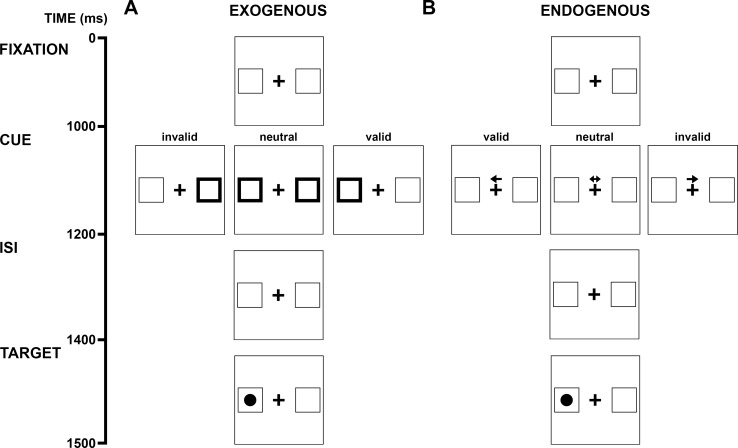
**Schematic illustrating the covert attention paradigm for exogenous (A) and endogenous (B) cueing conditions.** Participants were presented with a fixation screen consisting of a central cross flanked by two peripheral boxes, displayed for 1000 ms (0–1000 ms). A cue was then displayed for 200 ms, from 1000 ms after trial onset to 1200 ms after trial onset. Exogenous cues (A) were an increase peripheral box line width. Endogenous cues (B) comprised an arrow placed above the central fixation cross pointing toward the right or left peripheral box. Valid cues were congruent with the location of the subsequent peripheral target, whereas invalid cues were incongruent with the location of the peripheral target. Neutral cues provided were not indicative of the location of the peripheral target. Following cue presentation, the fixation screen was displayed for an inter-stimulus interval (ISI) of 200 ms (1200–1400 ms). After this, a circular target appeared in the right or left peripheral box for 150 ms (1400–1550 ms).

Covert spatial attentional orienting is typically investigated using a cueing paradigm [16], whereby participants are instructed to maintain stable gaze on a fixation point and respond as quickly as possible to the appearance of a peripheral target that is preceded by a cue. Crucially, the cue does not predict the target location, yet response times to targets presented in the cued location (valid cues) are typically faster than response times to targets appearing in uncued locations (invalid cues) [17] (Fig 1). This facilitation in detecting the cued target occurs because the cue reflexively attracts attention (exogenous attention), which in turn facilitates the processing of information at that location. Attention can also be oriented endogenously, and this form of attentional orienting can be measured using a similar task. Instead of non-informative peripheral cues, attention is oriented through centrally presented symbolic cues that inform participants of the likely target location. Participants are typically faster to detect targets that appear in the cued location than when they appear elsewhere, and this facilitation in detection occurs because of participants endogenously orient attention towards the cued location [18].

Two recent studies have shown that short bouts of intense exercise can affect shifts of covert attention within an exogenous cuing paradigm [13, 19]. Sanabria, Morales (13) initially reported no difference in facilitation toward targets presented a short interval after cue presentation (100 ms) between exercise and resting conditions. However, after closer inspection of their data they reported in a later paper that there was in fact a reduction in the magnitude of facilitation toward validly cued targets after exercise compared to rest [13, 19]. Using the same task, Llorens, Sanabria (19) replicated this finding, but only in low-fit individuals (mean VO_2_peak = 27.3 ± 6.2 ml∙kg∙min^-1^). To our knowledge, no studies have investigated the influence of fatigue associated with prolonged exercise on covert attention. The relative contribution of central fatigue can be largely influenced by the type of exercise performed. Prolonged exercise demands a high level of muscular and cardiorespiratory work, leaving potential for factors such as hyperthermia [20], substrate depletion [21], changes in cerebral energetics [1, 2] and decreases in synthesis and turnover of brain catecholamines [22]. Because covert attention can be affected by short bouts of exercise, it is reasonable to predict that the neural systems supporting this cognitive process might be vulnerable to the effects of central fatigue induced by an extended period of strenuous exercise.

Caffeine is a supplement commonly used by athletes as an ergogenic aid to combat the effects of exercise fatigue [23]. It is widely accepted that caffeine’s beneficial effects on physical performance and cognition are accomplished via its stimulant action on the central nervous system [24]. The question of how caffeine may influence covert spatial attention in the context of exercise fatigue is yet to be investigated. At rest, a moderate dose of caffeine (3–6 mg·kg^-1^ body mass) did not influence the orienting of attention within a cueing task [25] or the attention network test, which provides a measure of attentional orienting in addition to measures of alerting and executive control [26, 27]. However, caffeine did shorten response times across all conditions in the cueing task [25]. This is consistent with previous reports of caffeine improving performance on basic psychomotor tasks, such as simple and choice reaction tasks [28].

The aim of these two experiments was to investigate the effect of caffeine on endogenous and exogenous covert attentional orienting in the context of exercise-induced fatigue (the *Exercise* experiment) and sedentary rest (the *Rest* experiment). In the *Exercise* experiment, fatigue was induced using a demanding 3 hour cycling exercise protocol that is known to challenge central nervous system function [1, 2, 7, 21] while the *Rest* experiment consisted of 3 hours of sedentary activity. Endogenous and exogenous covert attentional orienting were measured using a cueing paradigm [16]. We tested multiple hypotheses. In the *Exercise* experiment, we predicted that exercise-induced fatigue would degrade both exogenous and endogenous covert attentional orienting. The second hypothesis was that the administration of caffeine would protect against the detrimental effects of exercise-induced fatigue on covert attentional orienting. We predicted that no changes in covert spatial attention at rest with, or without caffeine would be observed in the *Rest* experiment.

## Experimental Procedures

### Participants

Twelve physically fit participants (5 males, Mean VO_2_ peak 56 ± 6 ml∙kg∙min^-1^) with a mean age of 25 ± 9 (20–48) years volunteered to participate in the *Exercise* experiment. In the *Rest* experiment twelve physically fit participants (6 males, Mean VO_2_ peak 56 ± 7 ml∙kg∙min^-1^) with a mean age of 23 ± 2 (20–26) years volunteered to participate. Participants gave written informed consent and visited the laboratory on three (*Rest)* or four (*Exercise)* occasions to participate in a protocol conducted in accordance with the Declaration of Helsinki and approved by the University of Auckland Human Participants Ethics Committee.

### Experimental design

For the *Exercise* experiment participants received a dose of caffeine (7.5 mg·kg^-1^ body mass). This was administered in pill form within a double-blind, placebo-controlled, randomized cross-over design. Experimental trials involved 180 min of continuous cycling at a work rate equivalent to 60% of aerobic capacity with a minimum of 7 days between cross-over phases. Caffeine was administered over 2 doses–a 2.5 mg·kg^-1^ body mass dose before the exercise protocol, and a 5 mg·kg^-1^ body mass dose at 90 min into exercise. In the placebo trial, a placebo pill (maltodextrin) was administered before the exercise trial and at the 90 min time point in place of the caffeine doses administered in the caffeine trial. The *Exercise* experiment involved an additional randomized counterbalanced intervention—a psychostimulant drug included to test a separate hypothesis. These data are not included here and no trial order effects were detected that might confound interpretation.

Similarly, in the *Rest* experiment, participants received a moderate dose of caffeine (5 mg·kg^-1^ body mass) or placebo (maltodextrin) administered in pill form within a double-blind, placebo-controlled, randomized cross-over design. Participants completed two experimental trials involving 180 min of sedentary rest with a minimum of 5 days between cross-over phases. Caffeine was administered over 2 doses–a 2.5 mg·kg^-1^ body mass dose prior to the rest protocol, and a 2.5 mg·kg^-1^ body mass dose at 90 min.

Participants were asked to abstain from caffeine-containing items, such as coffee and tea, for the 24 hours before each experimental session. Caffeine doses for the *Exercise* and *Rest* experiments were selected based on similar doses employed in other studies for cognitive performance benefits at rest [26, 27, 29–31], and current recommended doses for physical performance benefits during exercise [32, 33]. This allowed for each experiment to explore the effect of a ‘typical’ dose of caffeine, in rest and exercise contexts, on covert spatial attention. Doses differed between experiments because the magnitude of the caffeine dose recommended to elicit ‘optimal performance’ is task dependent. For example, larger caffeine doses (5–9 mg·kg^-1^ body mass) are required in order to elicit an ergogenic effect during endurance exercise [32–35]. At rest lower doses are conducive to improvements in elements of cognition and mood such as visual information processing and alertness, which are desired performance benefits in sedentary settings (2–6 mg·kg^-1^ body mass) [26, 27, 29–31, 36]. Caffeine reaches a maximum plasma concentration approximately 1 hour after ingestion and has a half-life of 4 to 6 hours [8]. Dose timing was selected to ensure that intervention-associated changes in mood or arousal were possible over the course of the 3 hour rest or exercise period and to attempt to coincide the peak action of caffeine with post rest or post exercise measures. The timing of the doses resulted in the lack of a within-session caffeine free baseline for each experiment, hence the use of a placebo treatment.

### Preliminary Tests

At least 1 week prior to the experimental protocol participants performed a maximal cardiopulmonary exercise test on an electromagnetically braked cycle ergometer (Velotron Dynafit Pro, Seattle, WA, USA) to measure peak oxygen uptake. For the *Exercise* experiment, VO_2_max was estimated and used to prescribe a power output that required 60% VO_2_max for the experimental trials. Participants were familiarized with experimental protocols and the cueing task prior to the experimental trials. Due to a technical difficulty, a peak oxygen uptake measurement was not obtainable for one participant in the *Rest* experiment.

### Experimental protocol

Participants arrived at the laboratory at 8 am following a 12 hour overnight fast. Upon arrival, body mass was measured following voiding of the bladder for participants in the *Exercise* experiment. This was compared to a body mass measure collected post exercise in order to assess hydration status. In both experiments, participants were provided with a cereal-based breakfast, the quantity of which was self-selected on the first trial, and repeated for the remaining trials. With breakfast, participants received the first intervention dose in pill form (65 min before sedentary rest or exercise). Prior to exercise or rest (pre) participants completed the cueing task. The cueing task was performed as one task among a battery of six total visual tasks that examined the kinematics of basic eye movements, the results of which are not reported here. The visual task sequence was identical for all participants and therefore the additional tests did not introduce any systematic bias. The total time required to complete the cueing task was ~8 min, while the total test battery took ~50 min. After completion of the pre measures, participants then completed either 3 hours of sedentary rest (*Rest)* or cycled on an electromagnetically braked cycle ergometer (Velotron Dynafit Pro, Seattle, WA, USA) at a prescribed power output for 180 min (*Exercise)*. At 90 min into either protocol, participants received a second dose of the intervention in pill form. In the *Rest* experiment, participants were not permitted to eat during the experimental protocol, but were able to consume water as desired. In the *Exercise* experiment a carbohydrate solution (0.7 g carbohydrate·kg^-1^·h^-1^) was ingested at 15 min intervals during the cycling protocol in order to prevent substrate depletion and dehydration. Heart rate was monitored continuously throughout the experimental trials and was recorded at 15 min intervals using a heart rate monitor (FS1, Polar Electro, Kempele, Finland). Coincident with the recording of heart rate, participants’ mood and arousal was self-rated on visual analogue scales. The scales were composed of 25 centimeter lines with a related question above the scale and opposing descriptive statements at each end. For example, for the visual analogue scale assessing mood the descriptive question was, ‘how do you feel at the moment, what type of mood are you in’, while the opposite statements at either end were ‘very bad’–‘very good’. Participants rated their feelings by putting a perpendicular mark across each scale. Scores were marked in centimeters from the left of the line to the mark, such that a high score signified a good mood and high sense of arousal, while a low score represented a bad mood and low arousal. After rest or exercise, participants repeated the covert attention task (post). In the *Exercise* experiment a post measurement of body mass was obtained in order to assess fluid loss or gain as a result of the exercise trial.

### Measures of covert spatial attention

Covert attentional orienting was assessed using a Posner cueing task [16] with endogenous and exogenous cueing conditions. Visual stimuli (Fig 1) were presented on a cathode ray tube monitor (Philips 109S2; 1280 × 1024 pixel resolution; 85 Hz refresh rate), positioned at a viewing distance of 660 mm. At the beginning of each trial, a central fixation cross and two peripheral boxes located ± 10° laterally to the central fixation cross were displayed for 1000 ms. A visual cue was then presented for 200 ms. 200 ms after the cue was extinguished, a peripheral target (black circle, radius of 0.5°) appeared in the right or left peripheral box for 150 ms. Participants were instructed to maintain fixation on the central cross and to respond using a keyboard as quickly as possible when they detected a peripheral target to the left (left arrow key) or right (right arrow key) of fixation.

Endogenous cues consisted of a centrally presented arrow pointing to the right or left peripheral box. Arrows were centered 0.2° above the central fixation cross and subtended 0.5°. Exogenous cues consisted of an increase in the line width of a peripheral box from 0.15° to 0.20°. On valid trials, the target appeared in the cued location, whilst on the invalid trials it appeared in the opposite location. Neutral trials provided no cue indicating target location. In exogenous trials, the cue did not reliably predict the target location, with valid, neutral and invalid cues occurring with equal probability amongst the 90 trials. For endogenous trials, the valid cues correctly predicted target location 80% of the time thus, neutral cues were presented in 30 trials, valid cues in 48 trials, and invalid cues in 12 trials. The cue-target contingency was based on previous literature [16, 37, 38]. Neutral cues consisted of a double-ended arrow or an increase in line width of both peripheral boxes, respectively. Only responses occurring within 1000 ms of target onset were included in analysis.

In both the exogenous and the endogenous conditions, the targets appeared in the right or left peripheral box with equal probability. Keyboard responses (response time and left/right key) were collected using custom software written in Matlab (MathWorks R2010b, Massachusetts, USA) using the Psychophysics Toolbox extensions [39–41]. To ensure participants maintained fixation, eye movements were monitored with a head-fixed, 400 Hz eye tracker (ViewPoint Eye Tracker, Arrington Research Systems, Scottsdale, USA). Trials in which the eyes deviated > 1° from fixation were rejected from analysis.

### Data treatment and analysis

Repeated measures analyses of variance (ANOVA) were employed to explore the influence of the experimental interventions on covert spatial attention, heart rate, mood and arousal. Independent analyses were performed for *Rest* and *Exercise* experiments.

The final sample sizes for the *Exercise* experiment (n = 12) and *Rest* experiment (n = 12) satisfied *a priori* power analyses. For the *Exercise* experiment estimations of sample sizes were calculated with power set to 0.95, p < 0.05, a correlation among repeated measures of 0.62 and an expected effect size of 0.4. This calculation estimated that a sample size of 12 participants was sufficient to provide appropriate statistical power. Effect size and was derived from a previous study in which significant changes in saccade velocity were detected following prolonged exercise [7] while correlation among repeated measures was obtained from previous research citing a high correlation (0.62–0.97) across repeated measures for visual tasks [42]. Sample size for the *Rest* experiment was inferred from the calculation performed for the *Exercise* experiment.

Endogenous and exogenous response times and task performance (percentage of responses in the correct direction) in the covert spatial attention task were explored using a repeated measures ANOVA with factors 2 (*Rest*) or 3 (*Exercise*) INTERVENTION × 2 TIMEPOINT (Pre, Post) × 3 CUETYPE (Valid, Neutral, Invalid) for the endogenous and exogenous blocks respectively. A validity effect was calculated using the difference between valid and invalid response times, which reflects the time required to disengage covert attention from an invalid cued location and shift to the target location. The effect of our interventions on the validity effect was statistically explored using a repeated measures ANOVA with factors INTERVENTION × TIMEPOINT. Measures of heart rate, mood and arousal were explored using repeated measures ANOVA with factors INTERVENTION × 11 TIMEPOINT (15 min intervals, 15 min–165 min).

Though intervention allocation was counterbalanced, due to the repeated measures design of the experiment, there was a possibility of task learning and trial order effects confounding our measures of covert spatial attention. This was explored by using the same repeated measures ANOVAs described above, with the factor of TRIAL used in place of INTERVENTION.

Where relevant, main effects and interactions were explored using within-subject paired comparisons. The multiple comparison type I error rate was controlled using a false discovery rate criterion procedure [43]. In cases where sphericity could not be assumed, the Greenhouse-Geisser correction was used. Statistical significance was set at α = .05. Results are reported as mean ± standard error (SE), unless otherwise stated.

## Results

### Heart rate, fluid balance and subjective measures

#### Rest experiment

**Fig 2:**
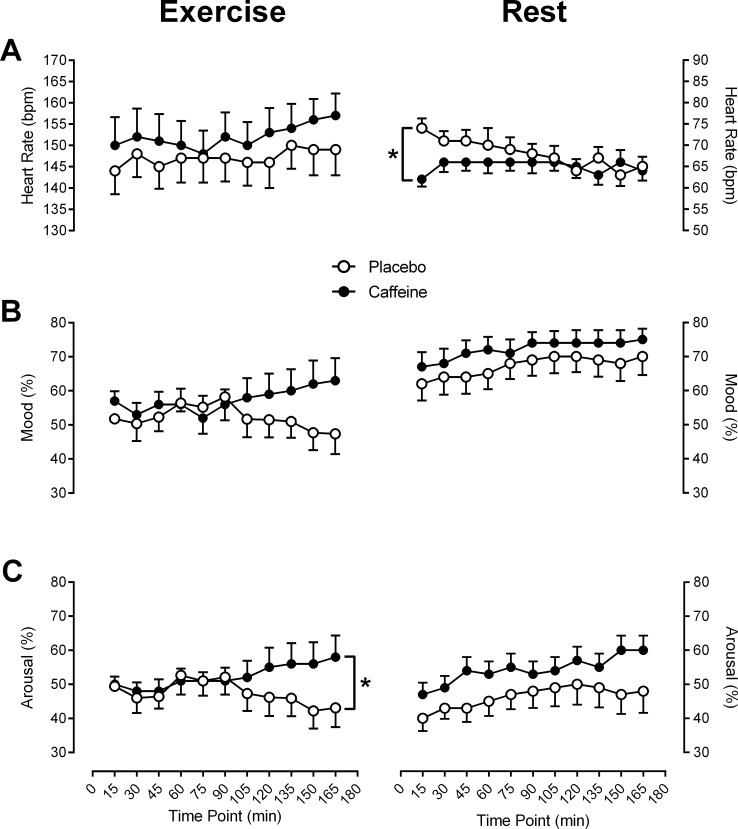
Heart rate, mood and arousal across the duration of the 180 min experimental trials for the *Exercise* experiment (left pane) and the *Rest* experiment (right pane). **These data were collected at 15 min intervals over the duration of the 180 min experimental trial.** (A) Heart rate for each intervention in beats per min (bpm). (B) Self-reported mood ratings for each intervention over the course of each experimental trial. Ratings from the visual analogue scale were converted to a percentage value whereby 100% represents a ‘very good’ mood, while 0% indicates a ‘very bad’ mood. (C) Self-reported levels of arousal over the duration of the experimental trial for each intervention. Levels of arousal were also converted to a percentage value where 100% indicates ‘very high’ arousal, while 0% indicates ‘very low’ arousal. Significance labelling in right panel A represents a significant difference in heart rate over time depending on intervention (interaction effect between intervention and time point), while left panel C represents a significant difference between caffeine and placebo (main effect of intervention). p < 0.05. Data represent mean ± SEM.

In the *Rest* experiment, heart rate (Fig 2, right panel A) differed significantly over the 3 hour rest period depending on intervention and time point (F_10, 110_ = 3.66, p < .01). This interaction appears to stem from higher heart rates at the onset of the rest period with placebo compared to caffeine. As the duration of the rest period continued, heart rate slowed to a rate similar to that observed in the caffeine trial. As illustrated in Fig 2 (right panel B), self-reported mood during *Rest* was not influenced by intervention (F_1, 11_ = 1.85, p = .202), but did improve over time point in both sessions (F_2.07, 22.7_ = 5.21, p < .05). Similarly, there were no significant effects of intervention (F_1, 11_ = 3.06, p = .108) on arousal. However, arousal did improve over the course of both sessions, as indicated by a main effect of time point (F_1.59, 17.5_ = 4.00, p < .05).

#### Exercise experiment

In the *Exercise* experiment, heart rate was not differentially influenced by intervention (F_1, 11_ = 4.80, p = .051), or by time point (F_2.76, 30.4_ = 2.94, p = .05), although there was a trend toward a main effect of time point on heart rate (Fig 2, panel A). Importantly, body mass did not differ pre to post exercise between the interventions. Measures of body mass were used to derive a measure of fluid balance for each experimental trial that quantified the extent of dehydration induced by the exercise bout. Fluid loss relative to body weight for placebo and caffeine was -0.19% ± 0.20 and -0.26% ± 0.26 respectively. There were no significant differences in fluid balance between interventions (F_1, 11_ = 0.21, p = .66). Furthermore, average levels of fluid loss did not exceed 2%–the point at which performance begins to decline due to dehydration [44]. Intervention did not differentially influence mood throughout exercise, however there was a trend toward a main effect of intervention (F_1, 11_ = 4.70, p = .05). These data are displayed in Fig 2, left panel B. Arousal levels did significantly differ during exercise depending on intervention (F_1, 11_ = 11.6, p < .01), with higher levels of arousal with caffeine (Fig 2, left panel C). There was a trend toward an interaction between intervention and time point, however this did not reach statistical significance (F_2.36, 26_ = 3.02, p = .058).

#### Summary

To summarize, at rest, the interventions did not differentially influence levels of self-rated mood or arousal. At the start of rest, heart rates in placebo were higher than with caffeine, but slowed to a similar rate 60 min into the rest duration. In the exercise trials, participants’ experienced equivalent physiological stress. Heart rate and fluid losses did not differ between interventions, and there was no dehydration. Self-rated mood was not differentially affected by our interventions, although arousal levels were heightened over the duration of the exercise with caffeine.

### Endogenous results

#### Error rates

In both experiments, endogenous error rates were not sensitive to task learning, with no trial order effects detected.

*Rest experiment*: Error rates were not influenced by intervention (*Rest*, F_1, 11_ = 1.50, p = .24) or time point (F_1, 11_ = 0.166, p = 0.69) in the *Rest* experiment. There was a trend toward a main effect of cue type (F_1.05, 11.6_ = 4.54, p = .05) on error rates, whereby lower error rates occurred in valid and neutral trials, and high error rates occurred in invalid trials, although this did not reach statistical significance. These data are reported in Table 1.

**Table 1:** Measures of Covert Attention. Data show response times and error rates as mean ± SD. Data were recorded before and after exercise (*Exercise experiment*) and rest (*Rest experiment*), respectively.

	**Placebo**				**Caffeine**			
	**Pre exercise**		**Post exercise**		**Pre exercise**		**Post exercise**	
***Exercise Experiment***	**Mean**	**SD**	**Mean**	**SD**	**Mean**	**SD**	**Mean**	**SD**
**Endogenous Condition**								
***Response Time (ms)***	**325**	**52**	**360**	**66**	**308**	**42**	**319**	**50**
*Valid Response Time (ms)*	301	49	329	71	288	39	294	48
*Neutral Response Time (ms)*	323	53	363	61	306	44	316	54
*Invalid Response Time (ms)*	349	62	388	74	330	50	348	57
***Error rate (% incorrect responses)***	**2**	**2**	**2**	**2**	**2**	**2**	**2**	**2**
*Valid error rate (%)*	1	2	1	2	1	2	0	1
*Neutral error rate (%)*	0	1	2	2	0	1	1	2
*Invalid error rate (%)*	4	6	3	5	5	6	5	5
**Exogenous Condition**								
***Response Time (ms)***	**325**	**50**	**372**	**59**	**321**	**46**	**325**	**51**
*Valid Response Time (ms)*	313	50	351	61	312	37	304	49
*Neutral Response Time (ms)*	325	51	381	65	320	59	333	54
*Invalid Response Time (ms)*	338	55	382	75	330	49	338	61
***Error rate (% incorrect responses)***	**2**	**3**	**2**	**2**	**1**	**2**	**2**	**1**
*Valid error rate (%)*	2	4	1	1	1	4	1	2
*Neutral error rate (%)*	2	3	2	3	1	2	2	2
*Invalid error rate (%)*	1	2	3	4	2	3	2	3
	**Pre rest**		**Post rest**		**Pre rest**		**Post rest**	
***Rest Experiment***	**Mean**	**SD**	**Mean**	**SD**	**Mean**	**SD**	**Mean**	**SD**
**Endogenous Condition**								
***Response Time (ms)***	**316**	**19**	**315**	**24**	**312**	**26**	**299**	**28**
*Valid Response Time (ms)*	305	26	295	25	302	21	284	34
*Neutral Response Time (ms)*	307	23	302	27	309	26	291	23
*Invalid Response Time (ms)*	337	28	347	30	327	40	321	37
***Error rate (% incorrect responses)***	**1**	**1**	**1**	**1**	**3**	**5**	**2**	**5**
*Valid error rate (%)*	0	0	0	1	0	1	1	2
*Neutral error rate (%)*	0	1	1	1	1	3	1	2
*Invalid error rate (%)*	1	3	2	4	6	12	5	12
**Exogenous Condition**								
***Response Time (ms)***	**330**	**27**	**331**	**24**	**330**	**25**	**312**	**36**
*Valid Response Time (ms)*	327	34	324	26	324	32	302	32
*Neutral Response Time (ms)*	313	23	323	35	320	20	313	43
*Invalid Response Time (ms)*	348	34	347	29	346	38	320	38
***Error rate (% incorrect responses)***	**1**	**1**	**1**	**1**	**0**	**1**	**1**	**1**
*Valid error rate (%)*	0	1	0	1	0	0	1	1
*Neutral error rate (%)*	1	2	1	2	1	3	1	3
*Invalid error rate (%)*	2	2	2	2	1	1	2	3

*Exercise experiment*: Similarly to *Rest*, error rates were not influenced by intervention (F_1, 11_ = 0.321, p = .58) or time point (F_1, 11_ = 0.031, p = .86) in the *Exercise* experiment. However, there was a main effect of cue type (F_2, 22_ = 20.0, p < .01). Within-subject paired comparisons revealed significantly lower error rates in valid compared to invalid trials (t_47_ = 4.80, p < .01), and lower error rates in neutral compared to invalid trials (t_47_ = 4.74, p < .01). However, there was no difference in error rates between valid and neutral trials (t_47_ = 0.281, p = .78).

#### Response times

*Rest experiment*: A repeated measures ANOVA revealed a main effect of time point (F_1, 11_ = 6.25, p < .05), and a significant effect of cue type (F_2, 22_ = 31.4, p < .01) on response times at rest. Furthermore, the difference in response times between time points depended on cue type, as indicated by a significant interaction (F_2, 22_ = 5.046, p < .05). Post-hoc comparisons revealed a significant decrease in response times following rest for valid (t_23_ = 2.62, p < .01) and neutral cues (t_23_ = 2.98, p < .01), but no difference in response times pre to post rest with invalid cues (t_23_ = -0.419, p = .68). There was no effect of intervention (F_1, 11_ = 1.67, p = .22) on response times.

**Fig 3:**
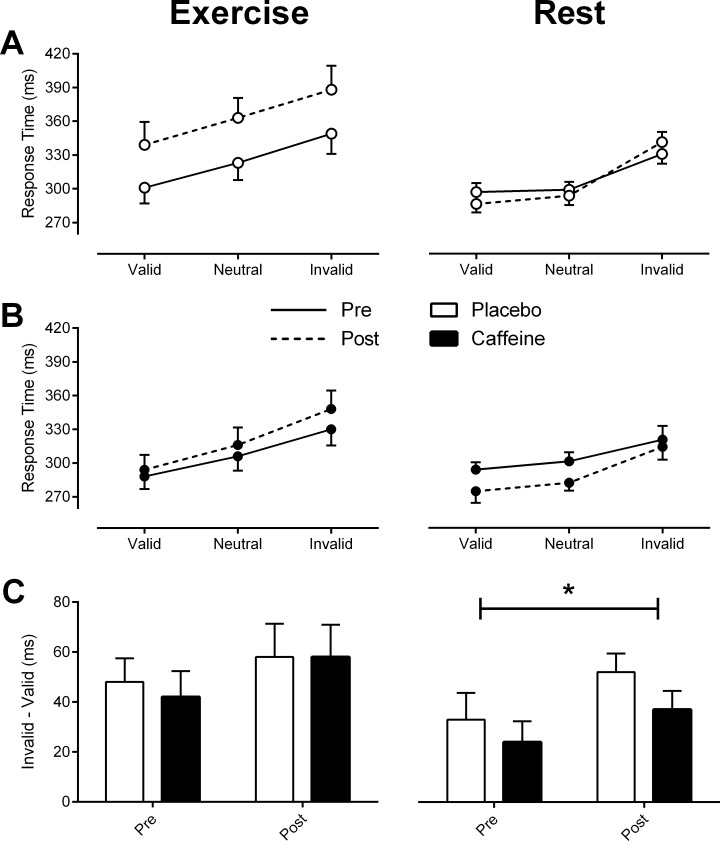
Measures of endogenous covert attention for the *Exercise* (left pane) and *Rest* (right pane) experiments. (A) and (B) Response time pre (solid lines) and post (dashed lines) exercise or rest across cue type for Placebo (white fill) and Caffeine (black fill). (C) Validity effect for each intervention (difference between invalid and valid cue types). Significance labelling in right panel C represents a significant difference pre to post rest in the magnitude of the validity effect (main effect of time point). Data represent mean ± SEM.

There was a significant increase in the magnitude of the validity effect after rest (Fig 3, right panel C) (main effect of time point: F_1, 11_ = 8.28, p < .05), but no effect of intervention (F_1, 11_ = 1.67, p = .22). This appears to result from participants responding faster to validly cued targets post rest compared to pre rest in both caffeine and placebo interventions. However, in the *Rest* experiment we did discover a task learning effect. A repeated measures ANOVA revealed a three-way interaction between cue type, trial and time point (F_2, 22_ = 3.70, p < .05). This stems from a small decrease in response times post rest to validly cued targets in the first trial, followed by a larger magnitude decrease pre to post rest in the second trial. The presence of this task learning effect confounds our ability to interpret the influence of our interventions on endogenous spatial attention, as the most likely explanation for the greater facilitation in response time toward validly cued targets is that, by the fourth presentation of the covert attention task (Trial 2, post rest) participants improved their response speeds simply by learning.

*Exercise experiment*: No task learning effects on response times were detected. As expected, response times were modulated by cue type (F_1.16, 12.8_ = 25.6, p < .01). Within-subject paired comparisons revealed that cueing influenced response times in a typical manner, with response times in invalid trials significantly slower than for valid trials (t_47_ = -8.98, p < .01). Response times were also significantly slower after 3 hours of exercise (main effect of time point: F_1,11_ = 5.39, p < .05) and differentially affected by intervention (main effect of intervention: F_1,11_ = 8.64, p < .05) (Placebo, 344 ± 15ms; Caffeine, 314 ± 10 ms). These results suggest that exercise fatigue increased (impaired) response times overall, but did not influence attentional orienting, since there was no interaction between cue type and intervention (F_2,22_ = 0.791, p = .47) or cue type, intervention and time point (F_2,22_ = 0.826, p = .45). This is also reflected in measures of the validity effect, illustrated in Fig 3 (left panel C). A repeated measures ANOVA on these data showed no main effects between the magnitude of the validity effect after 3 hours of exercise (F_1,11_ = 2.41, p = .15), in response to the drug interventions (F_1,11_ = 0.144, p = .71), or an interaction between time point and drug intervention (F_1,11_ = 0.149, p = .71).

*Summary*: Error rates were not differentially influenced by intervention in the *Rest* or *Exercise* experiments. At rest, response times to valid cues decreased across time point (in both interventions), leading to an increase in the magnitude of the validity effect. However, it appears that this modulation in attentional orienting can be explained by the presence of a task learning effect. A task learning effect was not present in the *Exercise* experiment, nor did intervention differentially influence attentional orienting. However, response times overall were significantly slower (worse) with placebo compared to caffeine.

### Exogenous results

#### Error rates

For both experiments, error rates did not display a task learning effect.

*Rest experiment*: Error rates at rest were unaffected by intervention (F_1, 11_ = 1.20, p = .30) or time point (F_1, 11_ = 1.18, p = .30). There was a main effect of cue type (F_2, 22_ = 3.44, p < .05), with significantly lower error rates following valid cues compared to invalid (t_47_ = 3.05, p < .01) and valid compared to neutral cues (t_47_ = 2.24, p < .05).

*Exercise experiment*: Similarly to *Rest*, error rates were unaffected by intervention (F_1, 11_ = 0.648, p = .44) or time point (F_1, 11_ = 0.022, p = .89). Unlike *Rest*, there was no significant differences in error rates due to cue type (F_2, 22_ = 1.50, p = .25). These data are reported in Table 1.

#### Response times

No task learning effects were detected in response times for both experiments.

**Fig 4:**
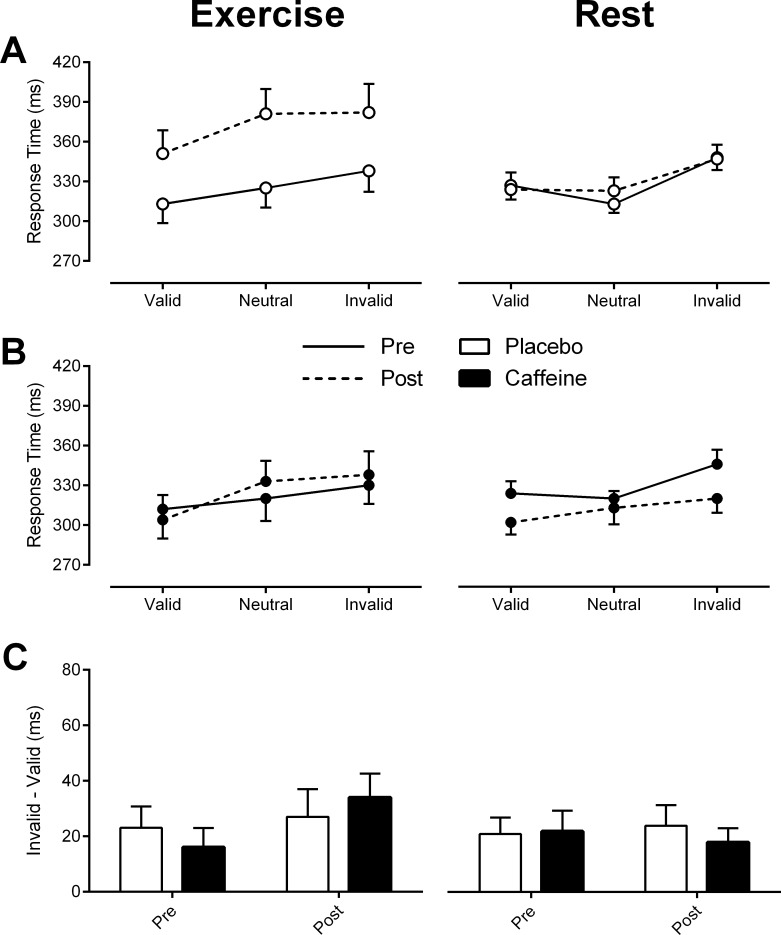
Measures of exogenous covert attention for the *Exercise* (left pane) and *Rest* (right pane) experiments. (A) and (B) Response time pre (solid lines) and post (dashed lines) exercise or rest across cue type for Placebo (white fill) and Caffeine (black fill). (C) Validity effect for each intervention (difference between invalid and valid cue types). Data represent mean ± SEM.

*Rest experiment*: Fig 4 (right panels A and B) illustrate response times across cue types for caffeine and placebo interventions. There were no main effects of intervention (F_1, 11_ = 1.25, p = .29) or time point (F_1, 11_ = 3.46, p = .09) on response times. However, there was an interaction between intervention and time point (F_1, 11_ = 5.57, p < .05), revealing that the extent to which response times shortened after rest depended on the intervention received. With Placebo, response times were similar between time points (pre rest = 329 ± 8 ms; post rest = 331 ± 7 ms respectively; t_35_ = -0.353, p = .73). Conversely, with Caffeine, response times decreased significantly from 330 ± 10 ms pre rest to 312 ± 10 ms post rest (t_35_ = 2.384, p < .05). In addition, the ANOVA revealed a main effect of cue type (F_2, 22_ = 14.23, p < .01). Post-hoc analyses revealed a typical cueing effect, with significantly faster response times to valid compared to invalid cues (t_47_ = -6.463, p < 0.01). As depicted in Fig 4 (right panel C), the magnitude of the validity effect was unaffected by time point (F_1, 11_ = 0.019, p = .89) or drug intervention (F_1,11_ = 0.254, p = .62), and there was no interaction between the two factors (F_1,11_ = 0.343, p = .57).

*Exercise experiment*: Similarly to *Rest*, response times in the *Exercise* experiment were also modulated according to cue type (F_2, 22_ = 7.23, p < .01). Post-hoc comparisons confirmed a typical cueing effect, with the response times for invalid trials significantly slower than that for valid trials (t_47_ = -6.13, p < .01). The repeated measures ANOVA also revealed a significant interaction between intervention and time point (F_1, 11_ = 8.65, p < .05), thus, the magnitude by which response times changed after 3 hours of exercise was modulated by intervention. Post-hoc comparisons revealed a significant slowing of response times pre to post exercise in placebo (t_35_ = -6.83, p < .01), but no difference in response times pre to post exercise with caffeine (t_35_ = -0.596, p = .56). These alterations in response time occurred generally, across all cue types. These results are illustrated in Fig 4 (left panels A and B). As observed in the endogenous condition, there was no change in the magnitude of the validity effect between time points (F_1, 11_ = 3.162, p = .10), or in response to intervention (F_1, 11_ = 0.028, p = .87). Furthermore there was no interaction between the two (F_1, 11_ = 2.34, p = .15). The validity effect across time point and intervention for the exogenous condition in the *Exercise* experiment is illustrated in Fig 4 (left panel C).

*Summary*: Overall, there were no effects of intervention on task performance in *Rest* or *Exercise* experiments. At rest, caffeine and placebo interventions did not differentially alter attentional orienting although response times overall did change after rest depending on the intervention received. Response times after rest were significantly faster in the caffeine trial, whereas with placebo response times were similar between pre and post rest time points. Attentional orienting was not influenced by fatiguing exercise with or without caffeine. However, overall responses times slowed significantly after exercise in the placebo trial, while this impairment in response times was prevented in the caffeine trial.

## Discussion

This study examined the influence of fatiguing exercise and caffeine on endogenous and exogenous covert spatial attention. Covert attentional orienting was unaffected by exercise-induced fatigue and the administration of caffeine. However, exercise fatigue impaired response time components of the attention task, an effect that was prevented by the consumption of caffeine. This finding suggests that the processes governing the pre-motor planning and execution of behavioral responses are sensitive to the effects of exercise-induced fatigue and modulated by caffeine, while the networks controlling covert attentional orienting are robust to both challenges.

Prolonged strenuous exercise had no effect on our measures of covert spatial attention. The magnitude of the validity effect pre to post exercise was unchanged for both endogenous and exogenous cue types, suggesting that the processes governing the covert attentional orienting are robust to exercise fatigue. This is surprising because the challenge to brain homeostasis associated with this exercise protocol is substantial. Others have shown that similar protocols significantly impair cerebral energetics [2, 21], and we have recently measured a reduction in the velocity of saccadic eye movements after same the exercise protocol [7], implicating fatigue related impairments in networks controlling the oculomotor system. Consistent with previous findings [7], there were no alterations in error rates in the covert spatial attention task after exercise, suggesting that response selection was robust to exercise-induced fatigue. However, the overall error rate was low because the task was designed to detect differences in response times between cueing conditions rather than changes inaccuracy. Therefore small changes in error rates would not have been detected.

Llorens, Sanabria (19) reported detrimental effects of short duration exercise on exogenous shifts of covert attention in low-fit (mean VO_2_max = 41.28 ± 6.32 ml∙kg^-1^·min^-1^) but not high fit (mean VO_2_max = 58.38 ± 2.96 ml∙kg^-1^·min^-1^) participants. Physical fitness may protect exogenous spatial attention during an exercise challenge, with well-trained individuals likely to show smaller exercise-induced reductions in cerebral metabolic resources [19]. The aerobic fitness of our cohort (mean VO_2_ peak = 56 ± 6 ml∙kg∙min^-1^) was comparable to Llorens, Sanabria (19) high-fit group. Therefore, we may have failed to observe an effect of fatiguing exercise as high fit individuals may be robust to the influence of exercise on covert spatial attention, despite other neural systems being compromised.

Although exercise fatigue did not alter the cueing effect in response to endogenous or exogenous cues, we did observe a significant slowing of response time. This effect of fatigue was most noticeable in response times to exogenous cues, where there was a significant slowing of response times post exercise across all cue types with placebo (Fig 4, left panel A). In the endogenous block, response times were significantly slower overall in placebo trials compared to the caffeine trials, but did not exhibit an interaction between intervention and time point. This suggests that exercise fatigue may have influenced the preparation and execution of motor responses (key presses). Accompanying the impairment in response times after exercise in the placebo trial were changes in self-rated arousal, particularly evident in the late stages of prolonged exercise. A general slowing of response time has been observed as a result of other forms of fatigue, such as sleep deprivation [45, 46]. Additionally, increased response time has been observed following prolonged fatiguing exercise with heat stress and dehydration [47]. In this study we used several strategies to avoid dehydration, heat stress or hypoglycemia. Fluid balance and euglycemia were maintained with a hydration and carbohydrate supplementation strategy and participants performed a set amount of work in a controlled environmental chamber. Despite these controls it is plausible that the neurohumoral and metabolic challenge of prolonged strenuous exercise [1, 21] exerted an influence on the speed of response time.

Caffeine prevented the slowing of response times observed after fatiguing exercise (Figs 3 and 4, left panel B). Decreases in reaction time and heightened arousal at rest following the administration of caffeine have been observed across several studies [30]. In addition, there is evidence to suggest that the improvements in reaction time with caffeine are greater in the presence of fatigue [29]. Interestingly, but not measured here, alterations in the central levels of dopamine and noradrenaline have been linked to the development of fatigue during exercise [48–50] and the maintenance of these central neurotransmitters through administration of caffeine or other psychostimulant drugs appears to attenuate the impact of fatigue on exercise performance [51] and the central nervous system [7, 52]. Therefore, caffeine may have preserved response times after exercise through its actions on central neurotransmission.

In the *Rest* experiment, endogenous and exogenous covert spatial attention was not differentially influenced by caffeine. There was no effect of 3 hours of rest on the magnitude of the validity effect in the exogenous cueing condition, nor by the administration of a moderate dose of caffeine (Fig 4, right panel C). Interestingly, there was a global decrease in response times across all cue types in exogenous trials after 3 hours of rest with caffeine (Fig 4, right panel B), while no differences between pre and post rest response times were seen with placebo. Because caffeine did not influence the validity effect, the global reduction in exogenous response times with caffeine is most likely related to an improvement in the preparation and execution of the motor response rather than an influence on the processes of covert attentional orienting. This finding adds to an already large body of literature demonstrating caffeine’s positive influence on the performance of basic psychomotor tasks [25, 29, 53–55]. Caffeine’s ability to influence psychomotor speed in this nature is most likely the result of the blockade in adenosine caused by caffeine, and a subsequent reduction in adenosine’s ability to inhibit dopaminergic activity in the basal ganglia circuits involved in voluntary skeletal motor output [8]. In addition the basal ganglia pathway involved in skeletal-motor output is functionally distinct from the parallel subcortical pathway involving the basal ganglia that is involved in oculomotor control and may contribute to orienting of visual attention [56].

In contrast to our observations at rest in the exogenous condition, in the endogenous condition we did observe a significant increase in the magnitude of the validity effect post rest, irrespective of intervention (Fig 3, right panel C). However, the observation of a task learning effect in the endogenous condition is the most likely explanation for the increase in the validity effect post rest across both intervention groups and limits further interpretation of these results.

Previous studies have described the presence of learning effects for several cognitive measures collected within a repeated measures experimental design [57, 58], but the sensitivity of endogenous covert attention to task learning is not commonly reported. We observed a significant decrease in response time to endogenous valid cues within each trial, with the largest pre to post decrease occurring in the second trial. Despite the use of a familiarization session to reduce the possibility of task learning, it appears that, at rest, participants improve their ability to interpret and respond to valid endogenous cues simply due to repetition of the task as a result of the experimental design. Given this, it may not be favorable to include endogenous cueing paradigms when employing a repeated measures design with acute interventions. Interestingly, in the *Exercise* experiment within-task learning effects were absent. Acute hypoxia, which also represents a significant challenge to cerebral homeostasis [59], appears to abolish practice effects in measures of cognition such as information processing and executive function [57]. Similarly, our results suggest that fatiguing exercise may eliminate endogenous task learning.

In sum, this study revealed that the covert orienting of endogenous and exogenous spatial attention is robust to the effects of fatiguing exercise and unaffected by the administration of caffeine. However, exercise fatigue globally impairs response times, irrespective of cueing condition or cue type, an effect that was prevented by caffeine. This finding suggests that the pre-motor planning and execution of the behavioral response prescribed by the covert spatial attention task are sensitive to the effects of exercise-induced fatigue. 3 hours of sedentary rest with caffeine had no differential effects on covert attentional orienting compared to placebo, but did speed response times overall. Interestingly, a within-task learning effect was observed at rest in the endogenous condition, highlighting an additional consideration when employing spatial cueing tasks in a repeated measures fashion.

## Supporting Information

S1 Dataset Exercise experiment dataset. (XLSX)

S2 Dataset Rest experiment dataset. (XLSX)
